# Middle East consensus recommendations on the use of young child formula (YCF) in toddlers

**DOI:** 10.1017/jns.2022.54

**Published:** 2022-07-08

**Authors:** Mohammed Al-Biltagi, Wafaa Faysal, Fatoumah Alabdulrazzaq, Hassan Alsabea, Ziad Bassil, Fadi Chamseddine, Imad Chokr, Ahmed El-Beleidy, Mostafa Ezzat, Antoine Farrah, Mohammad Mizyed, Ashraf Othman Saleh Sayed, Hussam Abu Talib, Yasser Wali

**Affiliations:** 1 Pediatric Department, Faculty of Medicine, Tanta University, Tanta, Egypt; 2 University Medical Center, Arabian Gulf University, King Abdulla Medical City, Manama, Bahrain; 3 Department of Pediatrics, Dr. Sulaiman Al Habib Hospital, Dubai, United Arab Emirates; 4 Department of Pediatrics, Al-Amiri Hospital, Kuwait City, Kuwait; 5 Pediatric Department, Medical Park Consultants, Abu Dhabi, United Arab Emirates; 6 Pediatric Department, Saint Joseph Hospital, Beirut, Lebanon; 7 Department of Pediatrics, American University of Beirut Medical Center, Beirut, Lebanon; 8 Pediatric Department, Al-Zahraa Hospital, Beirut, Lebanon; 9 Pediatric Department, Faculty of Medicine, Cairo University, Cairo, Egypt; 10 Pediatric Department, Dr. Hala Essa Bin Laden Hospital, Jeddah, Kingdom of Saudi Arabia; 11 Department of Pediatrics, Saint George Hospital Ajaltoun, Beirut, Lebanon; 12 Department of Pediatrics, Dr. Sulaiman Al Habib Medical Group, Riyadh, Kingdom of Saudi Arabia; 13 Department of Pediatrics, Dar Al-Shifa Hospital, Hawally, Kuwait; 14 Faculty of Medicine, Minia University, Minya, Egypt; 15 Pediatric Department, Primary Health Care Corporation, Doha, Qatar; 16 Child Health Department, Sultan Qaboos University Hospital, Muscat, Oman; 17 Department of Pediatrics, Faculty of Medicine, Alexandria University, Alexandria, Egypt

**Keywords:** Diet, Macronutrients, Malnutrition, Micronutrients, Vitamin D deficiency

## Abstract

The transition of foods during toddlerhood and the suboptimal diets consumed in the Middle East make children susceptible to malnutrition and micronutrient deficiencies. Based on international recommendations, coupled with the merits of clinical studies on the application of young child formula (YCF), a group of fourteen experts from the Middle East reached a consensus on improving the nutritional status of toddlers. The recommendations put forth by the expert panel comprised twelve statements related to the relevance of YCF in young children; the impact of YCF on their nutritional parameters and functional outcomes; characteristics of the currently available YCF and its ideal composition; strategies to supply adequate nutrition in young children and educational needs of parents and healthcare professionals (HCPs). This consensus aims to serve as a guide to HCPs and parents, focusing on improving the nutritional balance in toddlers in the Middle Eastern region. The panellists considere YCF to be one of the potential solutions to improve the nutritional status of young children in the region. Other strategies to improve the nutritional status of young children include fortified cow's milk and cereals, vitamin and mineral supplements, early introduction of meat and fish, and the inclusion of diverse foods in children's diets.

## Introduction

Toddlers (1–3 years of age) have high requirements of both macro- and micronutrients to support their rapid growth and immune development, alongside the development of body organs, including the brain^(1,2)^. While milk remains a basic food, toddlerhood is characterised by a transition from weaning foods towards a family diet, which may expose young children to nutrient inadequacy^(2)^. In addition, young children, especially those in developing countries and belonging to lower socio-economic strata, are prone to nutrient deficiencies and faltering growth rates due to inadequate amounts and/or poor quality of complementary foods^(1)^. The availability of data on the nutritional status of young children in the Middle East is limited. However, studies on school-age children in the region have shown that these children consume suboptimal diets deficient in nutrients like iron, calcium, zinc and vitamins A, C, D and folate^(3)^. The consumption of nutrient-dense foods (fruits, vegetables and milk) was found to be alarmingly low^(4,5)^, coupled with frequent, elevated consumption of fat (primarily saturated fat), sugar-sweetened beverages and energy-dense sweet and savoury snacks^(3)^ among Emirati children and adolescents^(4)^ and Saudi children^(5)^. Most consumed foods contain proteins, saturated fats and sugars at levels exceeding the daily recommended dietary allowances (RDA)^(3)^. Consequently, the dual burden of malnutrition, specifically calorific under-nutrition and childhood obesity, has arisen in the region and worldwide^(1)^. Malnourished children fail to grow and develop to their total capacity and are at an increased risk for non-communicable diseases (NCDs) like obesity and cardiometabolic diseases^(3,6)^.

In the Essential Nutrition Actions manual (2013), the World Health Organization (WHO) recommended using nutritionally adequate and safe complementary foods (like fortified foods and nutrient supplements) to complement breast-feeding and locally available foods to meet the dynamic nutritional requirements in infants and young children (>6 months), who do not achieve adequate nutrient intake through everyday foods^(7)^. International societies such as the European Food Safety Authority (EFSA), Nutrition Association of Thailand, Early Nutrition Academy (ENA), Federation of International Societies of Pediatric Gastroenterology, Hepatology and Nutrition, and European Society for Paediatric Gastroenterology, Hepatology and Nutrition have determined young child formula (YCF) to be one of several means to increase the intake of critical nutrients in young children for the reduction of nutrient deficiencies^(1,8–10)^.

YCF, also known as growing-up milk (GUM), are modified milk formulae specifically formulated for the nutritional needs of young children aged 1–3 years^(2)^. In certain cases, these formulae target specific sub-ages of toddlers, namely 1–2 years and 2–3 years of age^(11)^. These formulae are fortified with nutrients such as iron, vitamin D and essential fatty acids (EFAs) and contain less protein, sodium and saturated fats than cow's milk^(2,12)^. YCF is designed to support the nutritional needs of young children (from 1 year onwards) as part of a balanced diet^(13)^ and can be administered alongside breast-feeding. According to the EFSA, these formulae have ‘no unique role’ and ‘cannot be considered a *necessity* to satisfy the nutritional requirements of young children' compared with other foods that may be included in their regular diet^(11)^, including breast milk. However, clinical studies highlighted the positive benefits of YCF in toddlers by improvement of their dietary intakes and functional outcomes (which include various parameters of the child's physical and mental development and morbidity and mortality rates). Studies have indicated YCF to be associated with improved diet quality scores (PANDiet scoring system), better nutrient adequacy (predominantly micronutrients like iron and vitamin D, when used as a replacement to cow's milk)^(14)^, and increased feasibility to meet all EFSA-stated nutrient recommendations in young children^(13,15)^. In addition, there are data to demonstrate the benefits of YCF on gut microbiota and stool characteristics^(16)^, with reduced incidence of infections when supplemented with short-chain galacto-oligosaccharides/long-chain fructo-oligosaccharides (scGOS/lcFOS; 9:1) prebiotic mixture and *n*-3 long-chain polyunsaturated fatty acids (LCPUFA)^(16,17)^.

In light of clinical evidence and international recommendations around using YCF in toddlers, a group of fourteen experts from nine Middle Eastern countries established a consensus to improve dietary intakes and the nutritional status of young children across the Middle Eastern region through the supplementation of YCF.

## Methods

A comprehensive review of the existing evidence on the benefits of YCF in children was conducted, and several consensus statements were developed. The statements covered five major categories:

1. Relevance of YCF in young children (two statements);
2. Impact of YCF on nutritional parameters and functional outcomes (four statements);
3. Characteristics of currently available YCF and ideal composition for young children (two statements);
4. Other strategies to supply adequate nutrition to young children (one statement) and
5. Educational needs of parents and healthcare professionals (HCPs) (three statements).

These initial consensus statements were circulated to the expert panel members for voting. The expert panel consisted of fourteen paediatricians, paediatric gastroenterologists and neonatologists within the Middle East, each with at least 10 years of clinical practice experience. The panellists achieved the consensus through anonymous voting at a virtual meeting. Voting results were scored as ‘I agree’, ‘I disagree’ and ‘I would like to amend this’, and a consensus was considered when ≥80 % of panellists (more than twelve experts) voted ‘I agree’ on a particular statement. Following voting, an additional virtual meeting was held wherein the expert panel reviewed the voting responses and amended statements that did not achieve consensus. The advisors amended certain consensus statements, even if a consensus was reached, to customise them specifically for the Middle Eastern region.

## Consensus recommendations

### Relevance of YCF in young children

To determine the relevance of YCF in young children in the Middle East, the panel of experts summarised the critical points into two statements (Table 1), asserting the benefits of supplementing the daily diets of toddlers with YCF.

**Table 1.** Statements on the relevance of YCF in young children

Statement	Consensus (%)
Young children have high requirements of macro- and micronutrients to support their fast growth and development. In the Middle East, the diets of young children are often limited in essential micronutrients that include iron, calcium, zinc, folic acid, vitamin D, vitamin A and EFAs.	93
Excessive energy and protein intake should be avoided in young children due to their obesogenic effects and association with the development of NCDs later in life.	100

EFAs, essential fatty acids; NCDs, non-communicable diseases; YCF, young child formula.

### Impact of YCF on nutritional parameters and functional outcomes

A number of studies have demonstrated the improved nutritional intake upon implementing YCF. However, these studies have focussed on European countries (like Germany, the UK and France^(13,15,18,19)^), Australia^(20)^, New Zealand^(20)^ and Asia^(17)^. To specify the relevant criteria for toddlers in the Middle East, the experts compiled a series of four statements (Table 2). These statements address the indicators for the prescription of YCF and the quantity of YCF consumption.

**Table 2.** Statements on the impact of YCF on nutritional parameters and functional outcomes

Statement	Consensus (%)
YCF should be considered one of the solutions to effectively improve the nutritional status of toddlers and young children (1–3 years of age) in the Middle East and provide health benefits.	100
As part of a balanced diet, toddlers should drink 300–600 ml of milk or milk-based products daily. Substituting YCF for cow's milk can positively impact the nutritional adequacy of micro- and macronutrients in the diet of young children.	85
There is evidence that YCF supplemented with a specific mixture of scGOS/lcFOS (9:1) combined with *n*-3 LCPUFA or the probiotic *Bifidobacterium breve* M-16V can positively impact the gut microbiota composition and stool characteristics of toddlers aged 14–24 months and reduce the rate of infection in toddlers aged 11–36 months.	100
While we endorse the use of YCF in all toddlers and young children, it should be prescribed for children identified as being at nutritional risk during clinical evaluation.	85

scGOS/lcFOS, short-chain galacto-oligosaccharides/long-chain fructo-oligosaccharides; LCPUFA, long-chain polyunsaturated fatty acids; YCF, young child formula.

### Characteristics of the currently available YCF and its ideal composition for young children

One major challenge associated with YCF is that the composition of YCF varies from manufacturer to manufacturer. However, perusing available literature has shown that certain formulations may help achieve the recommended nutritional intake. The panellists have summarised this in two statements (Table 3).

**Table 3.** Statements on the composition of the currently available YCF and its ideal composition

Statement	Consensus (%)
The composition of YCF is not specifically regulated. Therefore, available formulae are not all created equal. A YCF formula with a composition that considers global recommendations for macro- and micronutrients and which does not contain sweeteners or taste modifiers should be preferred.	100
Per serving, YCF for children aged 1–3 years should provide the same amount of energy as whole-fat cow's milk (150 kcal/240 ml), 10–15 % of energy from proteins, 45–65 % of energy from carbohydrates, <8 % of energy from saturated fatty acids and <10 % of energy from added sugars. It should contain adequate amounts of LCPUFAs and avoid industrially produced *trans* fatty acids.	93

LCPUFAs, long-chain polyunsaturated fatty acids; YCF, young child formula.

### Other strategies to supply adequate nutrition to young children

The panel of experts explored alternative strategies (other than YCF) used for the supplementation of daily food in order to acquire the required levels of micro- and macronutrients. Their comprehensive search led to summarising beneficial strategies in the following statement (Table 4). Based on the consensus percentage, the panel felt it was prudent to amend the statement to provide clarity and to reflect their recommendation better.

**Table 4.** Statement on strategies to supply adequate nutrition to young children

Statement	Consensus (%)	Amended statement
Other strategies to improve the nutritional adequacy of the diet of toddlers and young children, such as fortified cow's milk and cereals, vitamin and mineral supplements, and early introduction of meat and fish intake, exist. However, these interventions often result in excessive energy, protein, and sodium intake, increased cost and low compliance.	65	Other strategies to improve the nutritional adequacy of toddlers’ and young children's diets include fortified cow's milk and cereals, vitamin and mineral supplements, and early introduction of meat and fish intake. However, these interventions *might* result in excessive energy, protein, and sodium intake, increased cost, and low compliance.

### Educational needs of parents and HCPs

Education and awareness are needed to resolve malnutrition risk (under-nourishment and childhood obesity). The expert panel summarised three critical statements (Table 5) to highlight the educational needs of parents and HCPs.

**Table 5.** Statements on educational needs of parents and HCPs

Statement	Consensus (%)
Educating parents and healthcare professionals about the indications, benefits and limitations of YCF is vital to improve awareness and increase adherence to nutritional recommendations, especially in the risk groups.	100
It is crucial to promote a balanced diet in toddlers and young children. Parents should be encouraged to offer their children a variety of foods to meet their nutritional requirements.	87
The benefits and the economic impact associated with using YCF should be discussed with the caregivers.	93

HCPs, healthcare professionals; YCF, young child formula.

## Discussion

The objective of the expert panel was to customise certain critical statements pertaining to the proper nutrient intake of toddlers in the Middle Eastern region. Recommendations from numerous global governing authorities (like WHO, EFSA and ENA)^(1,7–10)^ were reviewed and adapted to establish helpful guidelines for HCPs in the administration of YCF to supplement the daily diets of Middle Eastern toddlers. Furthermore, the panel focused on optimising and achieving the nutritional requirements of young children in the region.

### Relevance of YCF in young children

The WHO categorised countries, according to their micronutrient intake/status, into four groups, namely: (1) Countries in advanced nutrition transition; (2) Countries in early nutrition transition; (3) Countries with significant under-nutrition; and (4) Countries in a complex emergency^(21)^. Most countries in the Middle East belong to the first two categories. The Islamic Republic of Iran and Tunisia and all member countries of the Gulf Cooperation Council (Bahrain, Kuwait, Oman, Qatar, the Kingdom of Saudi Arabia (KSA) and the United Arab Emirates (UAE)), except Yemen, are countries in advanced nutrition transition with a high prevalence of overweight and obesity, and moderate prevalence of micronutrient deficiencies and under-nutrition. Egypt, Jordan, Morocco, Lebanon, Libyan Arab Jamahiriya, Palestine and the Syrian Arab Republic are countries in early nutrition transition with a moderate prevalence of overweight and obesity, moderate levels of under-nutrition in specific population groups, and widespread micronutrient deficiencies^(21,22)^.

Despite the presence of perennial sunshine, vitamin D deficiencies are prevalent in almost all age groups in the Middle East^(22,23)^. Studies have demonstrated that Lebanon, UAE and KSA had the highest prevalence of vitamin D deficits (45–62 %) among children with 25-hydroxyvitamin D (25[OH]D) ≤ 50 nmol/l^(22)^. Regarding iron deficiencies, as recently classified by the WHO, most Arab Middle Eastern countries fall within the category of moderate (20–39⋅9 %) deficiency^(24)^. In the Feeding Infants and Toddlers Study, the nutrient intake status of toddlers in the UAE revealed an inadequacy of iron intake (<70 % RDA) in 100 % of toddlers (12–23 months)^(22,25)^. Studies in school children revealed that 11⋅6 % of Saudi children and 39⋅6 % of Egyptian preschoolers suffered from anaemia. Observational studies in certain Middle Eastern countries have displayed 1⋅0 to 3⋅3/1000 live births with neural tube defects, highlighting a lack of dietary folate intake^(2,22)^.

Clinical trials have demonstrated the promotion of good general health accompanied by the reduced risk of chronic diseases from the sufficient intake of macro- and micronutrients derived from a combination of diet, fortified foods and supplements^(22)^. In France, a study compared cow's milk's nutrient intake and GUM in 118 toddlers (12–24 months). The results revealed that the use of GUM (>250 ml/d) significantly reduced the risk of insufficiencies in iron, vitamin C and α-linolenic acid, as compared to cow's milk^(26)^. In another study, an increase in YCF consumption and a decrease in cow's milk increased the likelihood of meeting the nutrient recommendations for young children in the UK^(13)^. Recently, a randomized German study demonstrated an elevation and improvement in vitamin D levels in children (2–6 years) using YCF (median daily intake of 234 ml) instead of cow's milk^(18,19)^.

### Impact of YCF on the nutritional parameters and functional outcomes

Exclusive breast-feeding for the first 6 months of life and continued breast-feeding up to 2 years and beyond, combined with the safe introduction of appropriate complementary foods, confer extensive and well-established benefits in infants and young children^(7)^. The experts advocated substituting cow's milk with YCF to improve the nutritional adequacy of macro- and micronutrients in toddlers’ diets. Several studies indicated the positive outcomes of replacing cow's milk with YCF in this age group. In a study conducted with approximately 90 Irish children (12–24 months), nutrient insufficiencies were observed in those consuming solely cow's milk. While the energy requirements, carbohydrates, proteins, dietary fibres and fats were congruent with recommendations, certain micronutrients (vitamin D and iron) were insufficient. The study showed that toddlers consuming GUM met the dietary requirements and reduced inadequacies for vitamin D and iron compared with solely cow's milk^(27)^. Furthermore, a multicentre, double-blind, randomised, placebo-controlled trial was conducted on growing-up milk ‘lite’ (GUMLi study; YCF fortified with micronutrients, reduced proteins and synbiotics) in Auckland and Brisbane. Of the 160 children (24 months), the ones who received GUMLi displayed healthy growth and favourable body composition (lower body mass index) as compared to those who received standard cow's milk^(19)^.

Studies have demonstrated the benefits of YCF supplementation with prebiotics, EFAs or synbiotics. It has been well-established that diet has a pivotal role in the composition and activity of gut microbiota. A study was performed to evaluate the effect of YCF supplemented with scGOS/lcFOS (9:1) and *Bifidobacterium breve* M-16V on developing the faecal microbiota in healthy toddlers. This randomised controlled clinical study included 129 children (12–36 months) from Thailand. The results showed that the supplementation of YCF positively influenced faecal microbiota development in toddlers and supported higher levels of *Bifidobacterium*. The synbiotic supplementation resulted in a more acidic intestinal milieu, producing softer stools^(16)^. In another study, the authors reported that the consumption of YCF (GUMLi), supplemented with scGOS/lcFOS (9:1) and LCPUFA (excluding *Bifidobacterium*), resulted in fewer infectious episodes in young children (80 % GUMLi *v*. 92 % GUM) as compared to cow's milk^(17)^.

The experts acknowledged the importance of YCF and endorsed its use in all toddlers in the region identified as being at risk of nutrient deficiencies. This requirement was established to maintain the Middle East consensus close to the EFSA recommendations, which state that ‘Fortified formulae, including young child formulae, are one of several means to increase *n*-3 PUFA, iron, vitamin D and iodine intake in infants and young children with inadequate or at risk of the inadequate status of these nutrients’^(8)^.

### Characteristics of the currently available YCF and the ideal composition for young children

Documented studies have elucidated that the composition of YCF varied from manufacturer to manufacturer. Thus, with the increasing importance of implementing YCF into the daily diets of toddlers, there is a need for standardised formulations. In 2015, recommendations for a follow-up formula for young children (FUF-YC) aged 1–3 years was proposed. According to this publication, FUF-YC should be given accompanying an age-appropriate mixed diet. With 1-2 cups (200–400 ml) of FUF-YC daily (approximately 15 % of total energy), carbohydrates should yield 9–14 g/100 kcal (>50 % from lactose), and other sugars (if present) should not be more than 10 % of the total carbohydrates. Protein levels from cow's milk-based formula should provide 1⋅6–2⋅7 g/100 kcal, and fat content should be 4⋅4–6⋅0 g/100 kcal. Calcium should provide 200 mg/100 kcal^(1)^. Similarly, in 2016, a report was published by an expert panel that provided nutrient recommendations for the composition of YCF for children aged 1–6 years. As per the manual, 2–3 servings (200 ml) of YCF were recommended for toddlers per day, with one serving aiming to provide 10–15 % of energy from proteins, 45–65 % from carbohydrates, <8 % from saturated fatty acids, <10 % from added sugars and a similar amount of energy as that of whole-fat cow's milk (150 kcal/240 ml)^(28)^.

Different countries may adapt the compositional requirements of YCF based on the nutritional status of toddlers in their region; however, the experts from the Middle East recommended following the recommendations advocated by WHO/Food and Agriculture Organization. Moreover, the panellists agreed that the ideal YCF composition should provide adequate amounts of macro- and micronutrients to the children without using artificial sweeteners, industrially produced *trans* fatty acids and taste modifiers, consistent with the global recommendations. The rationale for this consensus was confirmed by a recent study in Polish children (13–24 months) that evaluated their dietary preferences based on supplementation with YCF or standard cow's milk. The results showed that the introduction of YCF significantly elevated the consumption of sweetened dairy products, beverages and juice, with a decrease in plain fermented kinds of milk. There was evidence that the sweetness of YCF caused the toddlers to crave sweet-tasting foods. Those children on YCF with sucrose created an appetite boost and promoted hypersensitivity to sweetness in the toddlers. Contrastingly, those children consuming cow's milk had nearly 20 % lower daily carbohydrate intake, with levels in line with RDA, compared to the children consuming YCF^(29)^.

### Other strategies to supply adequate nutrition to young children

In some instances, the sole application of YCF supplementation to daily diet does not achieve the prescribed RDA levels, especially regarding vitamin D. When the daily diet does not fulfil the child's nutrient intake, EFSA endorses the use of other efficient fortified food alternatives and complementary feeding to maintain the nutritional balance in young children. Some examples included fortified cow's milk, cereals, cereal-based foods and supplements. In addition, the early introduction of meat and fish into complementary feeding in infancy and in the family diet in toddlerhood could be used to provide adequate nutrition to young children in the region^(8,14)^. It is noteworthy to mention that when cow's milk is used, the whole-milk version should be administered and not the skimmed milk version, as toddlers require the complete set of nutrients present in whole cow's milk. Skimmed milk is manufactured to target adults who desire to control their weight and thus contains lower fat levels. It is unsuitable for toddlers who require higher levels (than those in skimmed milk) of fat in their diet. Moreover, in cases of hypovitaminosis D, exposure of infants (wearing only a nappy) to approximately 30 min/week of sunlight is sufficient to elevate and maintain the recommended 25(OH)D >50 nmol/l levels of vitamin D^(23)^. However, one drawback is that these foods (YCF and other fortified products) may result in excessive intake of certain micronutrients and also impart an economic burden on the families, further leading to decreased compliance.

### Educational needs of parents and HCPs

The consumption of YCF may help infants and toddlers at risk of nutrient deficiencies to meet their nutritional requirements. Therefore, the experts concurred that it was essential to impart parental education to highlight the benefits of YCF in children alongside providing a balanced diet to meet their nutritional requirements. In many families around the globe, including the Middle East, data on the children's dietary intake clearly demonstrated the irregular food consumption patterns in this age group. Thus, to mitigate these problems, large-scale information campaigns would aid in creating awareness (regarding healthy eating habits and foods) among parents, caregivers and HCPs. Surveying available literature elucidated that family education and changing the lifestyle of many families would be necessary for young children to receive a balanced diet^(30)^.

The panel understood the importance of the added economic burden (of YCF supplementation) to the families in the region. A consensus to discuss this economic burden with caregivers was incorporated as the daily consumption of 300–600 ml of YCF could potentially incur an additional financial burden on the parents. Due to limited studies available on the economic impact of YCF on the average Middle Eastern family, it was unclear whether a healthy diet would be significantly more expensive than the other foods consumed by young children. In a recent study, Kuwaiti university students were surveyed regarding their consumption of fruits and vegetables. Analysis of the results revealed that consumption was low due to certain barriers like lack of awareness of benefits, the temptation of tastier processed foods, the short shelf life of fruits and vegetables, and time-consuming preparations^(31)^. Thus, the importance of educating families on healthy eating habits is a necessity.

### Limitations of the work

The limitations of this expert statement include the small number of experts (paediatricians, paediatric gastroenterologists and neonatologists) from whom the consensus was obtained. Nevertheless, the experts participating in this consensus are key opinion leaders within the field of paediatrics in the Middle Eastern region. Therefore, their opinions and best practices are valuable for improving the nutrient inadequacy amongst toddlers and young children in the region. Furthermore, other limitations of the expert statement involve addressing the course of action for difficulties related to the cost and availability of YCF in certain countries among specific groups belonging to the lower socio-economic strata of society. Finally, there are limited comprehensive studies and published data on the prevalence of malnutrition and micronutrient deficiencies in toddlers in the Middle East. Therefore, generalised statements rather than specific data have been included.

## Conclusion

The suboptimal diets of toddlers in the Middle Eastern region make them susceptible to micronutrient deficiencies and other NCDs later in life. Clinical evidence and international guidelines consider YCF to be one of the solutions to effectively improve young children's nutritional status. The group of fourteen experts has reached a consensus to copiously endorse the use of YCF in all toddlers (1–3 years of age) in the Middle East, with a particular focus on recommending YCF for children identified as being at nutritional risk during clinical evaluation. Such consensus will help HCPs and parents improve the nutritional imbalance in children in the Middle East. Moreover, further research on the nutritional status of toddlers in the region is required and will significantly help in fine-tuning the recommendations.
